# Towards Improving Hospital Managers’ Performance in Iran: History of a Pioneer Program Among EMRO Countries

**DOI:** 10.15171/ijhpm.2019.99

**Published:** 2019-12-08

**Authors:** Ali Maher, Mohammad Hossein Salarianzadeh, Abbas Vosoogh Moghaddam, Mehdi Jafari, Rouhollah Zaboli

**Affiliations:** ^1^Department of Health Policy, Economics and Management, School of Management and Medical Education, Shahid Beheshti University of Medical Sciences, Tehran, Iran.; ^2^Advisor to Secretariat of High Council for Health and Food Security, Ministry of Health and Medical Education, Tehran, Iran.; ^3^Secretariat of the High Council for Health and Food Security, Ministry of Health and Medical Education, Tehran, Iran.; ^4^School of Health Management and Information Sciences, Iran University of Medical Sciences, Tehran, Iran.; ^5^Health Managers Development Institute, Ministry of Health and Medical Education, Tehran, Iran.; ^6^Health Management Research Center, Baqiyatallah University of Medical Sciences, Tehran, Iran.

## Dear Editor,

Hospital managers and administrators are the key motivators for the success of each inpatient healthcare institution.^1,2^ Recent demographic and financial changes, rapid transformation in the healthcare systems, and a growing need for enhancing the healthcare quality have led hospital managers to increase efficiency and effectiveness of care delivery and patient satisfaction.^3^ Thus, the management of hospital has evolved over the years, and this is why training and development programs become increasingly important. Despite good intentions, training programs targeting hospital managers are only effective if grounded on the best practices. These training programs must be sustainable and continuously be monitored and updated.^4^

The present letter is a critique and a response to a letter recently published at the *IJHPM* entitled “*Capacity Building to Improve Hospital Managers’ Performance in West Asia.* ”^5^ This letter outlines the training and development program, targeting public hospital managers in Iran, initiated in 2016 by the Iranian Ministry of Health and Medical Education (MoHME) in collaboration with the World Health Organization (WHO). The purpose of this program is to empower public hospital managers, and to build capacity for improving their performance and productivity. Despite its efforts, the letter did not provide a comprehensive historical overview of the program. The letter also did not adequately acknowledge the intellectual property right of the project owners. There was no reference to the project owners responsible for delivering these training programs. Further, the title of the letter did not represent its content, which mainly covered Iran’s experience not the West Asia region.

The history of health managers’ professional development initiatives in Iran dates back to nearly two decades ago, in 1999, when some health managers participated in international training courses. Table shows a history of (in a chronological order) the main national and international training courses conducted.

**Table:** History of the National and International Management Training Courses

**Year**	**Principle Organization**	**Country**	**Agent**	**Target Group**	**Course Title**
1999	Management Development and Administration Reform Center of MoHME	Iran (Tehran)	Management Development and Administration Reform Center of MoHME	Health managers	Capacity Building of Health Managers
2000	Management Development and Administration Reform Center of MoHME	Thailand (Bangkok)	Chulalongkorn University	Top health managers	Capacity Building of Top Health Managers
2001	Center for Network Expansion and Health promotion of MoHME	Iran (Tabriz)	National Public Health Management Center	Health managers	Management Effectiveness Program (MEP)
2002-2007	Management Development and Administration Reform Center of MoHME	Sweden (Solna)	Karolinska University	Health experts	Karolinska Joint Scholarship Program for Health Management
2002-2005	Management Development and Administration Reform Center of MoHME	Germany (Heidelberg)	Heidelberg University	Health experts	Heidelberg Joint Master Program for International Health Management
2003-2005	Management Development and Administration Reform Center of MoHME	Iran (Tehran)	American University of Beirut in collaboration with WB, Harvard University, and WHO	Health system experts	Flagship Courses on Health Sector Reform and Sustainable Financing
2004-2006	Management Development and Administration Reform Center of MoHME	Germany (Karlsruhe)	Karlsruhe University	Supportive experts of health system	Facility Management in Health System
2005-2008	Management Development and Administration Reform Center of MoHME	Iran (Tehran)	Management Development and Administration Reform Center of MoHME	Health managers	ERAM Garden (Education, Research, and Advisement in Health Management)
2008-2010	Management Development and Administration Reform Center of MoHME	Iran (Tehran)	Industrial Management Institute	Hospital managers	Mini-MBA in Hospital Management

Abbreviations: WHO, World Health Organization; MoHME, Ministry of Health and Medical Education; MBA, Master of Business Administration.

Notwithstanding these academic events, with the beginning of the Health Transformation Plan in Iran in 2014, the need for managerial skills improvement in hospital managers was deeply felt. In 2015, a training assessment for hospital managers was done to identify their needs for inclusion in the planning, implementation, and evaluation of educational courses. Based on this assessment results, approximately 75% of the participants obtained low scores, and the qualitative analysis demonstrated their training needs. Consequently, the Hospital Managers Development Program was designed, and then started by the Hospital Management and Clinical Services Excellency Office in collaboration with Management Development and Administration Reform Center of MoHME, WHO country office of Iran, International Federation of Hospitals, and Iran University of Medical Sciences, Tehran, Iran. Also, International Relation Department of MoHME supported and facilitated the Hospital Managers Development Program initiative. In less than two years, all of the 600 public hospitals managers were trained in leadership and management skills,^6^ and WHO confirmed the proper implementation of this program.^7^ The tailored course entailed 28 days of training in 7 modules over a period of 8 months. This course was implemented in 7 management training centers in Iran. This successful experience in Iran, motivated the WHO to implement this course for health managers in Iraq and Afghanistan, which was held in the National Public Health Management Center in Tabriz, Iran. The WHO has supported the capacity building program since the starting point of the program, and in addition, involved in monitoring and evaluation of the training courses. In Iran, as a developing country, it will be important to continue conducting health management training, and also to justify the large investments in training programs, through rigorous assessment of their contribution to the capacity development of individuals, organizations, and health systems. To accomplish this important objective, the Health Managers Development and Training Supreme Institute, affiliated with the MoHME, was established in 2017, to organize and lead the professionalism, managers training, and management development in national and international health systems.

## Ethical issues

Not applicable.

## Competing interests

Authors declare that they have no competing interests.

## Authors’ contributions

All authors developed this letter and agreed upon the final manuscript.

## Authors’ affiliations

^1^Department of Health Policy, Economics and Management, School of Management and Medical Education, Shahid Beheshti University of Medical Sciences, Tehran, Iran. ^2^Advisor to Secretariat of High Council for Health and Food Security, Ministry of Health and Medical Education, Tehran, Iran. ^3^Secretariat of the High Council for Health and Food Security, Ministry of Health and Medical Education, Tehran, Iran. ^4^School of Health Management and Information Sciences, Iran University of Medical Sciences, Tehran, Iran. ^5^Health Managers Development Institute, Ministry of Health and Medical Education, Tehran, Iran. ^6^Health Management Research Center, Baqiyatallah University of Medical Sciences, Tehran, Iran.
